# Reconciling fragmented sectors of health research regulation: toward an ecosystem of processual regulation

**DOI:** 10.1057/s41599-021-01015-1

**Published:** 2022-01-07

**Authors:** Rhiannon L. Frowde, Edward S. Dove, Graeme T. Laurie

**Affiliations:** 1 School of Law, University of Edinburgh, Edinburgh, UK

## Abstract

The delivery of good outcomes from human health research is entirely dependent on the proper functioning of the attendant regulatory systems. This article focuses on the *processes* of regulation themselves, and how these might be better understood, so that regulators and other stakeholders have a strong normative basis upon which to pursue the regulatory objective of achieving outcomes with maximum social value. The argument is made that the concept of ‘processual regulation’—which promotes a whole systems approach to regulation —can assist greatly in the design, implementation, and review of human health research. This moves beyond the current often-fragmented approach to regulation towards a joined-up, reflective, and responsive system that has fitness-for-purpose at its core.

## Introduction

Health research has the objective of improving human health by understanding and improving current diagnostics and treatments or developing new ones (Institute of Medicine, 2009). Indeed, health research has the potential to produce significant benefit across a wide spectrum of activities, from providing a new understanding about disease trends and risk factors to revealing insights about outcomes of public health interventions, (in)effective patterns of care, and the (sub)optimal use of healthcare funds (Murphy and Topel, 2010; Grant and Buxton, 2018). The delivery of these outcomes, however, is entirely dependent on the proper functioning of the regulatory systems that govern the research endeavour. Not only must these systems facilitate the desired benefits, but they must remain constantly fit-for-purpose to respond to new research pathways and emerging ethical challenges. Regulatory systems are put under most strain in times of crisis and change, such as pandemics or other public health emergencies (Sigfrid et al., 2020).

The focus of this article is on the *processes* of regulation themselves and how we might better understand them so that regulators (among other stakeholders) can more effectively realise the regulatory objective of achieving outcomes with maximum social value. The contribution is to provide a strong normative basis to advocate for improved learning within the regulatory ecosystem that better supports this core objective. The argument is made that the concept of ‘processual regulation’—which supports and promotes a whole systems approach to regulation—can assist greatly in the design, implementation, and review of human health research. Such an approach moves beyond the current fragmented approach to regulation towards a joined-up, reflective, and responsive system of regulation that is also resilient in the face of ‘stress tests’ brought on by crisis and change.

The article proceeds as follows: Section “The current framework for health research regulation: a risk-based approach” gives a brief account of why the current approach to health research regulation is problematic in processual terms. Section “Whole system approach” responds to calls for a whole system approach (WSA), laying out for the first time what this might mean for the health research ecosystem while highlighting the limits of such an approach. Section “Processual regulation” provides a novel normative framework of processual regulation to complement WSA, demonstrating concrete ways in which the ecosystem might benefit and how challenges can be addressed. An advantage of processual regulation is that it does not necessitate immediate wholesale regulatory reform; rather, it suggests that for regulatory systems to keep up with the ever-changing nature of health research, the multiple, interconnected features of the entire ecosystem must be oriented towards the goal of social value and their roles assessed accordingly. The article concludes that the proposed approach can serve as a crucial first step towards creating a cohesive regulatory system that accords with the processual nature of health research itself.

### The current framework for health research regulation: a risk-based approach

Health research regulation can be defined as the ‘general ecosystem of activities, laws and regulations that seek to shape the conduct of any and all types of research involving human participants, or materials, data or tissue donated by them’ (Fletcher et al., 2020, p. 100). In most countries, the framework that operates is largely through a risk-based approach centred on identifying, assessing and mitigating risks to participants (NHS Health Research Authority, 2017, p. 4). But a collateral effect of this is a focus on defining, creating, and categorising ‘objects’ for regulatory attention, each bounded by its own regulatory rules calibrated by the nature and magnitude of perceived context-specific risks (McMillan et al., 2021, p. 8; Laurie, 2017; Vibert, 2014). To offer the United Kingdom as a typical example, ‘personal data’ are regulated by a data protection regime (Data Protection Act, 2018; UK GDPR, 2018), a ‘medical device’ is treated under a bespoke (European) regulatory regime, and this, in turn, is distinct from an ‘investigational medicinal product’ (Medicines and Medical Devices Act, 2021; The Medical Devices Regulations, 2002; Medicines and Healthcare products Regulatory Agency, 2020); ‘human material’ attracts specific legislative attention (Human Tissue Act, 2004; Committee of Ministers, 2016), while embryo research is the subject of highly specific and restrictive regimes in many countries (Human Fertilisation And Embryology Act 1990, as amended, 2008; Assisted Human Reproduction Act, S.C. 2004, c.2; The Research Involving Human Embryos Act 2002 No.145, 2002).

Although this regulatory approach is driven by the very important objective of identifying material risks and adequately protecting the rights of research participants, it does not align with the scientific perspective on what is valuable about the regulatory objects in question, namely, the core data that they contain. Yet, from a regulatory perspective, the pursuit of understanding the origin of the universe is a significantly more straightforward task than the search for the origins of a specific type of human cancer.

Complexity and potential confusion are further compounded by the fact that regulatory regimes at times overlap, causing researchers to experience difficulty in identifying, and complying with, an array of rules. This is especially problematic for highly innovative research that straddles various regulatory silos, as is the case for integrated biotechnologies (Quigley and Ayihongbe, 2018), embryonic stem cell research (Caulfield et al., 2009), and 3D bioprinting (Bicudo et al., 2020). Duplication of regulatory mechanisms can slow down, hinder, or even halt, important research or innovation (The Academy of Medical Sciences, 2016). A further consequence of this morass of sectors is that they can create redundancies in regulatory processes. This is especially true with the emergence of new health-related technologies, such as AI, and questions over how they should be regulated and by whom (Hoffmann-Riem, 2020). Because of a lack of ‘joined-up-ness’ and overarching regulatory oversight, each regulatory regime will—understandably—behove to its own requirements and new technology might be captured by multiple regulatory regimes. In sum, there are no coordinated means to triage emerging health research initiatives, let alone to identify and reduce system-wide redundancies.

As a result of all of this, a deep irony emerges: there is the serious risk of failing to fully protect and promote the core values and objectives of health research—namely, achieving improvements in human health while adequately protecting the human participant at the centre of regulatory attention (Fletcher et al., 2020, p. 100).

#### What is the regulatory problem seen in processual terms?

While a number of the above challenges are acknowledged in the literature, we do not yet have the means to think about the *systemic* problem in a sufficiently robust manner to pave the way towards a viable solution. A starting point, however, is to recognise the fundamental misalignment between fragmented regulatory approaches and the nature of human biology itself, on which all health research is founded. Biology is entirely characterised by processes, and the research consists of processes designed to understand said processes. If, then, we were to seek better alignment between research and regulation, we might reasonably ask: how far does *process* feature within regulatory approaches? By ‘process’ we adopt an everyday definition of ‘a series of things that are done in order to achieve a particular result’. For health research regulation, the end result is the production of ‘social value’ delivered without unduly compromising the protection of research participants (Council for International Organisations of Medical Sciences, 2017). The process of health research regulation is, then, the trajectory from initial research design through participant recruitment onto scientific analysis and towards the production of tangible social benefits. However, when looking for the process as part of regulation, three brief examples suggest that regulation currently occurs much more in ‘moments’ than a series of protective or promotional measures oriented towards a common end result.

Consent is a stalwart regulatory device used to protect research participants and to respect their autonomy. However, reliance on consent in human health research often involves a simple, upfront, one-off, binary offer/acceptance model, devoid of negotiation, deliberation, and iteration. As a result, much is potentially lost because this capturing of consent at a fixed time neither guarantees respect for participants’ autonomy over time nor does it ensure that regulatory arrangements are sensitised to actual or potential vulnerabilities of participants that can arise during research and from the mere fact of being involved the research endeavour (Brassington, 2021). While this is not to ignore alternative consent models—such as dynamic consent (Kaye et al., 2015; Budin-Ljøsne et al., 2017)—which attempt to empower research participants throughout their involvement in the research, the regulatory burdens of giving effect to this are considerable, e.g. ensuring that participants are adequately informed at all stages to support their right to withdraw. Moreover, such a model perpetuates a fixation with consent as the principal regulatory device in health research regulation rather than asking whether, where, and how consent might fit into the ecosystem as part of a holistic enterprise.

In a similar vein, anonymisation is a common technique that is used in data-driven research to protect participants’ privacy interests, often as an alternative to consent. However, it is often viewed simply as a technical solution applied to data to reduce the risk of re-identification when data are used and linked. As with many approaches to consent, anonymisation usually occurs at a distinct stage or moment in the research trajectory. The risk here is that this can perpetuate an assumption that ‘anonymised data’, once so achieved in status, are thereafter safe to use for research and beyond the scope of regulatory frameworks. This fails to recognise, however, that data may shift between anonymity and identifiability depending on how they are used and shared in the course of the entire research endeavour. Thus, downstream recipients of anonymised data might proceed on a misapprehension when, in fact, the linkage of data to their own datasets might lead to the identifiability of participants with whom they have no relationship whatsoever. This is a particularly strong concern in Big Data projects (Sethi and Laurie, 2013; Laurie et al., 2015; Lowrance, 2003).

Finally, a further regulatory moment concerns ethics approval. Whether styled as a Research Ethics Committee (REC) or an Institutional Review Board (IRB), in all instances ethics bodies are charged with the scrutiny of research projects before they begin, assessing whether they are ethical and comply with relevant rules, principles, and standards. That is, these bodies play a crucial role at the ex-ante stage of research and some have gone so far as to label RECs the ethical ‘gatekeepers’ (Singh and Wassenaar, 2016). In reality, however, these bodies are relatively constrained in their ability to follow researchers—and participants—across the research lifecycle. Generally, RECs and IRBs have limited resources and the capability to monitor research beyond the preliminary stage of review (Hedgecoe, 2012; Denneny et al., 2019). Commonly, these bodies remain only tangentially connected to an approved research project, such as when a project needs to submit a ‘substantial amendment’, i.e. a material change to an aspect of the project. It is potentially problematic, then, that a gap emerges where researchers and research participants alike may feel an absence of ongoing support (from RECs or other regulatory actors).

The risk-based regulatory model, the phenomenon of regulatory silos, and the manifestation of regulatory ‘moments’ all illustrate the lack of ‘joined-up-ness’ of the current health research regulation ecosystem. This is not to deny or overlook sterling efforts on the part of many regulators in the last few decades. In the UK, for example, the Health Research Authority has a responsibility to ‘lead’ the various regulatory authorities and coordinate their functions, including those across the UK’s four nations, but it can only do so much as multiple different regulatory authorities exist, each with their own remit and ‘objects’ to regulate.

The question arising from all of this is whether, and if so how, might these fragmented sectors and regulatory ‘moments’ be better joined up. In the next section, we identify that recent calls have been made for a ‘whole systems approach’ and we explore what this might require.

### Whole systems approach

The problematic consequences of the current regulatory environment have been recognised by the Academy of Medical Sciences, which called for a ‘whole systems approach’ to address these challenges (The Academy of Medical Sciences, 2016, p. 5). The terminology of a whole systems approach is increasingly prevalent in healthcare literature, for example, in informing public health approaches (Rutter et al., 2017; Bagnall et al., 2019; Luke and Stamatakis, 2012). However, there has been little work to date on how such an approach might be realised within health research regulation. Accordingly, this section sets out what it means to take a whole systems approach and it makes the case for the benefit that this can bring to health research regulation.

#### What comprises a whole systems approach?

Whole systems approaches (WSAs) function as a social learning process, responding to complexity through a flexible way of working. This is achieved by bringing together stakeholders in a given system to develop a shared understanding of emerging problems and integrating action to bring about sustainable long-term change (Stansfield, South and Mapplethorpe, 2020).

WSAs are based on the appreciation that change is a complex, non-linear process, caused by many interconnected influences, both distal and proximal. Any complex process necessitates an approach that captures the whole picture, recognising diverse influences, rather than considering events discretely in a piecemeal fashion (Government Office for Science, 2021, pp. 78–89; Hawe et al., 2009; Rowe and Hogarth, 2005). This is achieved by considering the nature of the relations of different elements of a system, explaining how these interact and cause particular outcomes. However, because these interactions are continually shifting as a result of stimuli both within and outside the system, it is necessary to continually re-consider these interactions. Obesity systems are an example: there is a web of causative factors that are inextricably interconnected. These include one’s genetic makeup, societal factors that influence stress, sleep, or diet, and wider environmental factors such as any public health structures that are in place (or absent). The extent to which any single event is causative is unpredictable, for this is subject to vagaries of change (Public Health England, 2020; Government Office for Science, 2021; Bagnall et al., 2019). Consequently, WSAs require ongoing and reflective cycles of learning, with reforms delivered through incremental steps and in collaboration with stakeholders over the long term. Ongoing (but not incessant and overly frequent) reflection and adaption is thought to enable the delivery of a ‘whole’ understanding of complex processes, unlike traditional models where reflection comes only at the end of a process (Garside et al., 2010). Therefore, key elements of a WSA are:

- ‘*Complex systems thinking*’: recognition that elements of a system are influenced by a complex, ever-changing network of interconnected components.
- *Collaborative action*: developing an understanding of complex components of a system requires the active involvement of all stakeholders to form a collective understanding of the issue, context, and wider system.
- *Learning culture*: the ever-changing nature of complex systems requires ongoing feedback methods, such as embedding monitoring and evaluation processes, enabling effective long-term action.

WSAs have been conceptualised in several forms in the literature, with no ‘best answer’ emerging as to how to achieve whole systems change. For example, the Vanguard method focuses on the ‘end to end’ flow of a system, that is, focusing on the flow of work through a system rather than individual functions, as seen in the design and management of the Munro Review of Child Protection (Seddon, 2005; Gibson and O’Donovan, 2013; Munro, 2011); the McKinsey 7S model focuses on the alignment of different elements of a system, requiring consideration of all the variables, which has been used to inform the Munro Review but also healthcare organisational design strategies (Munro, 2011, p. 152; Northamptonshire Healthcare NHS Foundation Trust, 2017, pp. 8–10); and a Soft Systems Methodology focuses on accommodating different perspectives and priority of stakeholders, seen in the Drug Policy Commission’s report on legal highs and in healthcare research (Birdwell et al., 2011; Augustsson et al., 2019).

A common feature, however, is to ensure that fitness-for-purpose remains under constant review. Creating a collective understanding of moments in a system and incorporating this when determining responses not only promotes effective regulation but also creates outcomes that can be supported by all stakeholders because they are likely to reflect their views or input (or at least can demonstrate that these were taken into account). Involving stakeholders throughout the process can create shared meaning and purpose, and this can enable actors across the system to understand how it operates and their role within it (Attwood et al., 2004; Baud and Mackenzie, 2007; Hawe et al., 2009). A major challenge identified in implementing a WSA is that shifting to such a system may appear chaotic at first, for traditional understandings that systems ought to be linear are deeply embedded (Attwood et al. 2004; Baud and Mackenzie, 2007; Rowe and Hogarth, 2005). However, a WSA is not a short-term fix, but rather a long-term solution to effective systems change, and therefore this initial chaos is a necessary consequence of a longer-term commitment.

How might a whole systems approach be of value for health research regulation? As identified earlier and elsewhere (Grant and Hood, 2017; Greenhalgh and Papoutsi, 2018; Aron, 2020), health research is a complex system; and, seen through the lens of a Whole Systems Approach the ‘moments’ in this system cannot, and should not, be viewed in isolation because they are inter-connected. Events that happen within one area of the health research process affect, and are affected by, those that happen in other areas. It follows that some regulatory tools might be inadequate when considered across the entire trajectory. Thus, a WSA can offer significant benefit in understanding how various elements in an ecosystem operate and potentially interact; in turn, adopting a WSA can promote more reflection and ‘joined-up-ness’. However, with no ‘best approach’ emerging from the WSA literature, it is insufficient simply to posit that a WSA should be adopted. More particularly, from a regulatory perspective and when considered against the ethical values at stake within an ecosystem, there is a need to complement any whole system approach with an appropriate normative framework for establishing a WSA within health research regulation. Not only will this provide a solid and legitimate basis upon which any WSA can be built, but it can also help to promote buy-in if the ethical foundations are made explicit and the edifice makes sound sense in terms of regulatory theory and practice.

We contend in the next section that a processual approach to regulation has the potential to provide the necessary normative basis for such a framework.

### Processual regulation

Processual methodologies are often used to study organisational, social, and political change. These enable effective responses to addressing necessary change by requiring regulation to reflect the experiences of actors and the environments within which the process takes place (Dawson, 2005; Taylor-Alexander et al., 2016). Common to WSAs, change is characterised by complexity (Dawson, 1994; Abbott, 2016), but the particular contribution of a processual approach is that it starts from the notion that these complex systems are processes in which change is an inevitable component. And, rather than attempting to determine the cause of any change, processual approaches propose that it may only be understood by looking at the elements and stages of the process itself: that is, the events and actors involved (Abbott, 2016). Thus, processual approaches are focused on explicating the logic of process, rather than its outcomes (Sewell, 2005, p. 2).

Processual approaches question the value of long-term planning and rules, focusing on emerging processes of learning and adapting rather than foresight (Whittington, 2001). Importantly, a processual approach is not premised on the expectation of change, nor does it aspire to wholescale reform. Rather than attempting to force change, a processual approach works with processes as they currently are, in a flexible, adaptive manner. This may take the form of following existing regulations and encouraging stakeholders in a system to bring forward their views and experiences to promote system-wide learning and to co-produce *incremental* change where there is sufficient consensus that this is merited.

Pang and colleagues have suggested that such a learning system for health research should have four components: (i) a defined vision for the system; (ii) clearly identified research priorities; (iii) the setting and monitoring of ethical standards; and (iv) the monitoring and evaluation of the system itself. They see stewardship as a central feature of such a system (Pang et al., 2004). We agree and go further: the processual regulatory experience itself must be captured as part of this, that is, the lived experience of all involved. This must necessarily mean that each of these four components can themselves be brought under scrutiny, lest the defining of a vision or setting of standards become ‘fixed’ in the regulatory space and driving more compliance and control behaviours rather than a genuine openness to learning. For us, a processual approach requires ‘the temporal-spatial examination of regulatory spaces and practices as these are experienced by all actors, including the relationship of actors with the objects of regulation’ (Taylor-Alexander et al., 2016, p. 175). Most importantly, this kind of engaged approach to regulation *over time* is well placed to capture the range of ethical values that are at stake within the given enterprise. Reflection and review shift from particular stages or moments to the entire trajectory of activities. This re-orientation helps to ensure that the full range of ethical considerations are taken into account in deliberating whether, where, when, and how change might occur.

The value of this kind of approach to processual regulation is best illustrated by revisiting the regulatory moments identified earlier in this article.

#### Implications for consent, anonymisation and ethics review

The roles of consent and anonymisation have been outlined above. Indeed, often these are deployed as alternative tactics in conducting health research, i.e. either researchers seek consent *or* they anonymise participant data. On either approach, there is a belief that sufficient regulatory attention has been given to the protection of participants’ interests (Academy of Medical Sciences, 2016). However, when seen holistically as part of a WSA, it becomes apparent that this model neglects the temporal and spatial dimensions of the subjects’ experience of becoming a research participant (Laurie, 2017, p. 63). For example, a research participant might, over time, grow dissatisfied with the focus that research is taking: research findings might give rise to an increased risk of discrimination, including against a group that involves the original research participants. Consent cannot protect against this (Stoljar, 2021). At best, consent in the form of refusal means that participants can opt out. But this does not address wider ethical concerns; moreover, it can jeopardise the research itself. Similarly, while the anonymisation of individual-level data might protect against (some) privacy threats, it cannot address any similar longer-term wider concerns.

In both examples—consent and anonymisation—there is a risk that the putative commitment to prioritising the interests of participants cannot be adequately discharged because once the regulatory object is obtained from a subject, e.g. data or tissue, the subjects are often no longer (sufficiently) involved in the process of research or research regulation. This overlooks their continuing interest in the objects in question and indeed in the research itself (McMillan et al., 2021). The point here is not to deny the value of either consent and anonymisation in the right circumstances. Rather, it is to highlight a central risk in the risk-based approach that is currently adopted in health research, namely, setting up regulatory regimes around participant expectations about their autonomy and privacy that often cannot be met. A WSA approach that took a holistic perspective would not reduce the ethical issues simply to concerns about autonomy and privacy. Processual regulation suggests that mechanisms for ongoing engagement can be very important to capture and reflect the potential changing attitudes of participants.

Moreover, if new risks to participants arise as research progresses—or if there are reasons to imagine that other ethical concerns are at stake—then a WSA founded on robust processual regulation would suggest that feedback loops be built into the system to support and consider further ethical reflection. This could, for example, lead back to an enhanced role for RECs or IRBs to track research across the regulatory lifecycle. That said, it might fairly be objected that this is not a proper role for ethics bodies who are more ‘gatekeepers’ than ‘stewards’. But, in much the same way that Pang et al. (2004) foresee a role for stewardship, so too it has been argued that regulatory stewardship can—and should—be a more integrated part of the regulatory design (Dove, 2020). Cast as ‘… guiding others with prudence and care across one or more endeavours—without which there is a risk of impairment or harm—and with a view to collective betterment’ (Laurie et al., 2018), regulatory stewardship is a role that might emerge when a holistic view of health regulation is taken. Irrespective of its particular form, this would be a tangible instantiation of processual regulation that is not currently an explicit part of the ecosystem. A light-touch version of this is the call that has been made for clinicians to be more involved in the work of ethics bodies to share experiences and inform the practice of oversight more generally (Kolstoe, 2019).

#### Social value

A whole-system approach, driven by processual regulation, would focus on the end result of the endeavour. In the case of human health research, this is social value. But in what ways might a processual regulatory approach re-orient how social value is viewed or pursued? For one thing, it would put into perspective the regulatory moments set out immediately above. Thus, for example, consent and anonymisation would be valued *relative to* their role in securing ultimate social value. In some cases, consent or anonymisation might be deemed unnecessary or even illegitimate faced with a sufficient pressing need for data or research of significant social value. Consider the October 2021 joint report of the UK House of Commons Committees on Health & Social Care and Science and Technology into lessons learned from the HM Government’s early response to the COVID-19 pandemic. This was damning about the failures in data sharing and a culture of caution about privacy driving behaviours even when faced with a global crisis (House of Commons, 2021). This suggests that a system-wide assessment is required with respect to the full range of priorities in play. A WSA—with social value at its centre—can promote this.

Multiple instances of social value can drive processual regulation as part of a WSA (Ganguli-Mitra et al., 2017). These include (i) pursuit of the ongoing trust and input of research participants, (ii) demonstration to researchers that the regulatory system will support and respond to their needs, (iii) creation of ‘safe spaces’ to capture learning moments when mistakes or missteps do arise and which can be folded back into the ecosystem through feedback loops, and (iv) recognition that social value can arise even if the science does not achieve its intended purpose. An obvious example of this last instance is negative findings arising from research, e.g. when a hypothesis is not proven. Instituting mechanisms to fund and publish such findings has considerable social value in showing that some avenues of research are no longer worth pursuing. More generally, (v) social value can be generated by meaningfully and respectfully involving participants and the public in research. This can generate goodwill and might serve to ground future social licence for new and productive research.

The above examples only touch the surface, but at present regulatory regimes simply do not require the mapping or evaluation of the range of potential social values that emerge from any given research initiative (van Delden and van der Graaf, 2021). The analysis in this article suggests that it is of crucial importance to do so not only to maximise the utility of the research and the efficacy of the regulatory regime but this can be promoted and supported through a whole system approach and operationalised through processual regulation.

#### Further instances of processual regulation in health research regulation

Other works have advanced a processual approach in specific sectors, such as embryo research or reliance on the public interest to legitimate health research (McMillan, 2021; Sorbie, 2020).

Consider, for example, how the law in relation to human embryo research fixes regulatory objects such as the ‘frozen embryo’ or the ‘research embryo’ and imposes the 14-day rule for lawful research in many countries. However, this fails to reflect the biological developmental processes in play and ignores the ongoing interests of embryo donors in how their embryos might be treated in research. Taking a WSA that sought to capture the full range of interests, a human embryo used for research would remain an entity of moral significance connected to (in meaningful ways) the persons who created and donated it. Adopting a processual approach would, in turn, allow for recognition of this and perhaps ground calls for embryo donors to have more say in embryo research (McMillan, 2021).

A further example of a processual regulatory approach is found in Sorbie’s account of the public interest (Sorbie, 2020). This concept sits at the heart of much data-driven research and is a legal device used to authorise the use of data for research purposes. However, its meaning and scope remain highly elusive. It is recognised that—as a regulatory tool—it is not based on any particular set of values. A WSA would recognise this and would promote the dynamics of legal and social frameworks within which public interest decisions could be iteratively constructed. Using the example of the Confidentiality Advisory Group (CAG) in England and Wales (which assesses whether the duty of confidentiality can be lifted such that researchers and other actors can lawfully use patient information without patient consent), Sorbie has demonstrated that a processual approach enriches understandings of public interests over time by engaging with ‘… the messy realities and subjectivities, both of the law, as broadly conceived, and of evidence of actual publics in a pluralistic society’ (Sorbie, 2020, p. 261). A body like CAG helps to ‘close the loop’ for the entire system by publishing its decisions on individual research applications, thereby fleshing out common understandings of how the public interest is being understood and applied across a range of research initiatives. It is an instance of processual regulation in action.

#### The likelihood and limits of a processual approach to health research regulation

The contribution of a processual approach to regulation is the underlying understanding that laws, regulations, and rules should be open to flexible interpretation, discussion, and (re)evaluation. This is not synonymous with arguing for a change to these. Rather, the normative aim is to create a framework where regulations are open to reflection and revisitation based on the lived experience of putting them into action. This will enable them to be operationalised—or adapted as needs be—with a view to best accommodating the range of interests at stake, as revealed through a WSA. At a systems level, there are at least two ways in which this processual approach—linked to social value—can be delivered:

- For individual projects, the regulatory trajectory becomes something to be navigated together by all stakeholders, finding the best way through and identifying and promoting the range of social values that arise as the research proceeds;
- For the health research regulation ecosystem, instances of best practice can be captured from individual projects and folded into learning opportunities for future initiatives by the use of regulatory feedback loops (Sethi 2019).

Consequently, a processual approach will not necessarily dispense with current risk-based, nor with regulatory tools like consent or anonymisation; but equally it has the potential to do so. A WSA captures the full range of interests and implications of committing to these relative to the ultimate goal of the pursuit of social value, broadly conceived.

As recognised above, regulatory systems are under most strain in times of crisis, such as public health emergencies, and this is a valuable final example of where a processual approach can confer significant benefit. As the COVID-19 pandemic worsened through 2020 and beyond, the social value of research into vaccine development increased exponentially. This meant that some research that would have previously fallen below the value thresholds for ethics approval, such as ‘human challenge’ trials exposing participants to the SARS-CoV-2 virus, was approved. Although the laws relating to health research in most countries did not change, regulatory bodies took action with a notable shift away from a highly cautious approach focussed on safety and efficacy. This has not come without a degree of strong scepticism in some quarters; indeed, the regulatory approach has necessarily been piecemeal because the response was time-critical. Little was in place at a social-structural or regulatory level to promote lesson learning, as the recent UK House of Commons report highlights all too starkly (House of Commons, 2021). For the future, however, there is now no excuse for failing to put in place robust learning mechanisms. The approach offered in this article can provide a crucial first step.

This said the limits of any such approach must be recognised. On the plus side, this is not a plea for wholescale reform, indeed it is quite the opposite. It is a proposal that recognises that change is inevitable and incremental. It is accepted, nonetheless, that some of what is being re-imagined has inevitable economic and transactional costs, e.g. what would regulatory stewardship involve, who would carry this out, and who would fund it? More fundamentally, however, it is important to appreciate that what is being advocated is a way to look at current systems with fresh eyes: (i) we need not see the roles of consent and anonymisation in an atomistic fashion; (ii) we can promote and support debates about what counts as social value without huge expense, and (iii) training of regulators, researchers, and members of ethics bodies can be re-oriented in light of system-wide learning (Sykes, 2021; Charlebois and Labrecque, 2007). Securing a commitment to systems learning can enable regulation to both safeguard the rights and interests of the human subject and to realise scientific aims and maximise social value. A processual approach to regulation can embrace a wide array of human practices that make up health research and move towards a means to co-produce regulation without first determining what the outcome might be.

## Conclusion

In this article, we have argued that the current risk-based system of health research regulation is insufficiently responsive to the ultimate objective of realising social value from human health research. Taking a Whole System Approach that is based on mutual learning from all stakeholders, novel understandings can emerge about the efficacy—or otherwise—of key components in regulation, such as the respective roles and value of consent, anonymisation, and ethics review. We advocate the value of processual regulation in giving effective to this learning. A pro-cessual approach promotes on-going evaluation of the guiding principles, rules, and tools of regulation *relative to* the main regulatory objective. This supports regulatory systems that can adapt incrementally. Wholesale reform is not what is needed. Equally, a more holistic approach can accommodate a much richer understanding of social value than is currently reflected in regulation practices. Practically, regulators might implement feedback loops—continuous cycles of learning, adaptation, and training—to improve the efficiency of regulation. Processual regulation can better align oversight mechanisms with health research practices themselves paving the way to help maximise the overall benefits that result from human health research, for researchers and participants alike.

## Data Availability

Data sharing not applicable to this article as no datasets were generated or analysed during the current study.
